# Development and Validation of the Youth Sociopolitical Action Scale for Social Media (SASSM)

**DOI:** 10.1007/s40894-023-00208-w

**Published:** 2023-03-01

**Authors:** Sara Wilf, Laura Wray-Lake

**Affiliations:** Department of Social Welfare, Luskin School of Public Affairs, University of California - Los Angeles, 337 Charles E Young Dr E, 90095 Los Angeles, CA USA

**Keywords:** Adolescence, Emerging adulthood, Scale development, Sociopolitical development, Technology, Online activism

## Abstract

Youth sociopolitical action, which encompasses a broad range of behaviors to dismantle systems of oppression, is increasingly taking place on social media and digital platforms. This study presents the development and validation of a 15-item Sociopolitical Action Scale for Social Media (SASSM) through three sequential studies: in Study I, a scale was developed based on interviews with 20 young digital activists (*M*_*age*_=19, 35% cis-gender women, 90% youth of color). In Study II, Exploratory Factor Analysis (EFA) identified a unidimensional scale using a sample of 809 youth (M_age_=17, 55.7% cis-gender women, 60.1% youth of color). In Study III, an EFA and Confirmatory Factor Analysis (CFA) were used to confirm the factor structure of a slightly modified set of items with a new sample of 820 youth (*M*_*age*_=17, 45.9% cis-gender women, 53.9% youth of color). Measurement invariance testing was conducted by age, gender, racial and ethnic background, and immigrant identity, confirming full configural and metric invariance, and full or partial scalar invariance. The SASSM can further research on youths’ efforts to challenge oppression and injustice online.

## Introduction

Young people have dramatically increased their sociopolitical action online with the advent of new platforms and tools (Jenkins et al., 2016). Social media is a new context and space for sociopolitical action, similar to schools or community centers, where young people are building movements that change norms and policies at national and global levels (Anyiwo et al., 2020) and can transcend structural barriers to political participation, such as the voting age (Marchi & Clark, 2021). Scholars have called for additional research exploring how young people are taking sociopolitical action online (Anyiwo et al., 2020), yet the field lacks a validated scale to capture online sociopolitical action for youth holding diverse age, gender, racial and ethnic, and immigrant identities. This study uses qualitative and quantitative methods to develop and validate a scale to measure youth sociopolitical action on social media.

### Conceptualizing Online Sociopolitical Action

Sociopolitical action is derived from sociopolitical development theory (SPD), which proposes developmental processes whereby youth become aware of systems of oppression, gain agency, and take liberatory action (Watts et al., 2003). SPD was developed from the ideas of Black activists and scholars, other liberatory scholars including Freire’s (2002) conceptualization of critical consciousness, and from insights derived from the lived experiences of Black boys in the U.S. Although SPD may be applicable to youth across the globe, it has largely been studied in the U.S. context (Heberle et al., 2020). Three mutually reinforcing dimensions are central to SPD: critical social analysis, in which youth learn about the root causes of systems of inequity; political efficacy, in which youth become empowered to create sociopolitical change; and sociopolitical action, where youth resist oppressive systems and take action towards liberation (Watts et al., 2003). Sociopolitical action focuses on resisting oppression, and thus centers behaviors that change policies and norms for marginalized groups at individual, group, or societal levels (Watts et al., 2011). Whereas the first two dimensions of critical social analysis and political efficacy are psychological processes, the third dimension of sociopolitical action constitutes behaviors to advance social liberation (Watts & Hipolito-Delgado, 2015). SPD posits that all three dimensions are positively correlated with one another (Watts et al., 2003), which is also supported by quantitative research (Diemer et al., 2017; Hope et al., 2020). In particular, critical social analysis has been associated both with in-person political engagement (Diemer et al., 2017) and online civic actions (Bañales et al., 2020).

Given its open forum for expression and lower barriers to access than other civic spaces (Marchi & Clark, 2021), social media offers a unique space for young people to resist oppressive systems and pursue justice. Thus in order for the field to comprehensively understand youth’s SPD, sociopolitical action in a social media context must be systematically studied. This study defines online sociopolitical action as young people’s *individual, relational, and collective efforts on digital platforms and social media to expose and rectify systems of oppression and inequity*, based on SPD theory and inspired by Ballard and Ozer’s (2016) definition of activism as well as scholarship on youth resistance to oppression (i.e., Aldana et al., 2019; Anyiwo et al., 2020; Hope et al., 2016). This definition encompasses young people’s efforts both to reflect upon past injustice and to imagine or create new futures for themselves and their communities (Ginwright, 2010). For example, on social media youth challenge dominant narratives (Gross, 2017), engage in public education campaigns (Erlick, 2018), create positive collective social identities (Kelly, 2018), offer emotional support to peers facing racism and discrimination (George Mwangi et al., 2018), and participate in mass actions like tweet storms to support social movements (Carney, 2016). Artivism, a combination of art and activism, is often incorporated into youth sociopolitical actions on social media to resist oppression and imagine new norms and narratives (Perera et al., 2021). For many young people, sociopolitical action on social media is inextricably intertwined with in-person sociopolitical actions to support social movements (Wilf & Wray-Lake, 2021). For example, the Movement for Black Lives used social media to call out interpersonal and structural racism, provide psychological support, and mobilize millions across the globe for in-person protests and voting (Carney, 2016). Amid spikes in hate crimes against Asian Americans during the onset of COVID-19 in 2020, young people utilized social media to call out anti-Asian racism and organize local protests and community support events (Cao et al., 2022).

### Existing Measurement of Youth Online Sociopolitical Action

There has been an increase in scales developed to measure young people’s critical consciousness (i.e., Diemer et al., 2017, 2022; McWhirter & McWhirter, 2016; Thomas et al., 2014). Critical consciousness is a framework closely related to SPD that includes the same core dimensions, albeit with sometimes different terminology. These critical consciousness scales were developed to measure youth behaviors that challenge systems of oppression in person rather than social media, with items such as “Contacted a public official by phone, mail, or email to tell him/her how you felt about a social or political issue” (Diemer et al., 2022). Such items leave out ways that youth could contact public officials on social media sites such as Twitter and Instagram. Some items in these scales could occur both in-person and on social media, such as “I work to make sure that people are treated equally and are given equal chances” (Thomas et al., 2014) or “I have participated in demonstrations or signed petitions about justice issues” (McWhirter & McWhirter, 2016). However, because young people’s response to these items may or may not include actions on social media, it would be impossible for researchers to more closely examine the relationship between youth sociopolitical action on social media and other developmental processes.

Other recently developed measures of sociopolitical action were informed by SPD and critical consciousness, but are not specific to social media. Notably, the Anti-Racist Action Scale was developed guided by SPD and critical consciousness (Aldana et al., 2019), and the Resistance and Empowerment Against Racism (REAR) Scale also measures anti-racist actions (Suyemoto et al., 2022). The Anti-Racist Action Scale includes items that could occur in person or on social media, such as the item “Challenged or checked a friend who uses a racial slur or makes a racial joke” (Aldana et al., 2019). Although the Anti-Racist Action Scale does not include any items mentioning social media, the REAR scale includes one item pertaining to social media: “I publicly respond to other’s online postings about racial discrimination” (Suyemoto et al., 2022). Similar to critical consciousness measures of action, the items in these scales (with the exception of the one noted above) do not explicitly note where anti-racist actions are taking place, thus making it difficult for researchers to isolate youth sociopolitical action occurring on social media.

Several studies have measured youth online political participation, but with notable limitations (e.g., Bañales et al., 2020; Jones & Mitchell, 2016; Velasquez & La Rose, 2015; Wray-Lake & Sloper, 2016; Yankah et al., 2017). First, most of these scales measure individual-level rather than interpersonal or mass actions, such as a variant of the question asking how often youth “express your opinion online regarding a political issue” (Velasquez & La Rose, 2015) or “read social and/or political posts on social networking sites” (Yankah et al., 2017). Second, these scales were not developed in line with sociopolitical development theory and, correspondingly, were not explicitly focused on youth behaviors online to resist oppression. For example, the Online Social Activism Scale was developed “to measure an individual’s participation in online social networking behaviors specifically related to social and/or political views/issues” (Yankah et al., 2017, p. 67). Finally, there are documented differences in how youth are civically engaged online by gender (Brandtzaeg, 2017), racial and ethnic identity (Auxier, 2020), immigrant identity (Zimmerman, 2016), and age (Yankah et al., 2017), yet these scales are not validated with these groups.

## The Current Studies

Young people are using a variety of strategic behaviors on social media to counter oppression and enact new liberatory futures for themselves and their communities. Although quantitative research has utilized study-specific scales to measure similar concepts as sociopolitical action on social media, such as online political participation, there is a need for a scale developed with an anti-oppressive lens based on sociopolitical development theory. This study, which is composed of three sequential studies, developed and validated the Sociopolitical Action Scale for Social Media (SASSM) with a gender, racially and ethnically, and immigrant diverse sample of young people, as well as youth from adolescence (14–17) to emerging adulthood (18–25). Although young people around the world are engaging in sociopolitical action on social media and their actions to challenge oppression may be similar to those used by young people in the U.S., this study focused on U.S.-based youth for two reasons: the initial qualitative study upon which the scale was based was conducted with U.S. based digital activists, and most research using SPD theory has been conducted in the U.S.

### Study I Method

#### Participants and Procedure

Participants for Study I, a qualitative study, were youth (*N* = 20) ages 16–21 (M_*age*_ = 19) who were highly civically engaged on Twitter and resided in 10 U.S. states. Youth were recruited through the “followers” of social movement chapters on Twitter, including March for Our Lives, Sunrise Movement, and Black Lives Matter. Although participants were not asked to identify their political ideology, it is likely that most participants identified as liberal. We return to this issue in the discussion to further consider how the sampling shapes the interpretation of findings. Participants were contacted through Twitter direct message and invited for one-hour semi-structured phone interviews, which were conducted between March and September 2020. Fourteen (70%) participants were first or second generation immigrants, 10 (50%) identified as part of non-majority religions in the U.S., 45% identified as cis-gender men, 40% as cis-gender women, and 15% as gender nonbinary. Participants identified as Asian American (45%), Black (20%), Latinx (5%), Middle Eastern and North African (10%), Multiracial (10%), and White (10%).

#### Study I Findings and Item Development

Please refer to Wilf and Wray-Lake (2021) for a full accounting of the methodology and themes in Study I. Critical consciousness, a framework that is closely related to sociopolitical development theory (Diemer et al., 2021), was used as a sensitizing concept in analysis. Using inductive Constant Comparative Analysis (Fram, 2013), the authors identified three forms of sociopolitical action on social media (which were called online civic engagement) that formed the basis for items in this scale: Restorying, Building Community, and Taking Collective Action. Each of these forms of sociopolitical action were described by youth as ways to resist and heal from the psychological effects of oppression. Restorying, where youth reframed, challenged, and imagined new narratives, included four sub-themes:(1) personal storytelling, (2) challenging and reframing dominant narratives, (3) envisioning new futures, and (4) self-love. Building community included (1) emotional support and (2) allyship. Youth described emotional support as individual (self-care), relational (providing support to others), and collective (group healing). Taking collective action included (1) holding people accountable and (2) artivism. All youth viewed holding people accountable as an important way to make their voices heard in the face of structural barriers to political participation (such as the voting age). Fewer youth mentioned artivism, but those who did explained that it was a powerful tool for resistance and healing.

Based on how young people in Study I described their online civic engagement, this study conceptualized youth sociopolitical action on social media as behaviors that transcend one-time, discrete actions (like using a hashtag or signing a petition), an approach which allows for a range of actions and is more resistant to technological advances. Because youth are participating in so many distinct actions online to create sociopolitical change, a scale measuring youth sociopolitical action through these distinct types of actions could include hundreds, if not thousands, of items. For example, a young person might debate a sociopolitical topic by posting their own opinion, posting a news article, sharing someone else’s post, commenting on someone else’s post, creating a video, using a hashtag — the list goes on, and will continue to increase with the growth of new platforms and tools. Indeed, youth sociopolitical action on social media is constantly evolving with new technologies, which could render such a scale rapidly obsolete. To remedy this issue, the present study aimed to measure youth higher-level actions such as telling their personal story on social media to challenge stigmas and stereotypes, rather than discrete actions such as signing a petition. This approach aligns better with young people’s own descriptions of their online sociopolitical action (Wilf & Wray-Lake, 2021).

Items developed for the scale based on the abovementioned themes were identified in the interviews (see Table 1 below for how each theme and sub-theme translated into items). First, items were created based on each theme and sub-theme, without limiting the number of items. Next, items were reduced or merged to reduce overlap. For example, two items focused on holding people accountable (differentiated by individuals and institutions) were merged. Whenever possible, item wording reflected exact terms used by youth in the interviews. The initial item set included items focusing on specific actions (such as “participate in a digital strike or protest for a social or political issue”), but these were removed before EFA analysis due to the scale’s focus on higher-level sociopolitical actions. Given that building community was not interpreted in the same way by all youth, items that reflected the sub-themes of emotional support and allyship were included instead of an item on community building.

For the SASSM, respondents report the frequency of engagement online by answering, “How often do you do the following on social media?” Responses were a 5-point frequency scale based on a monthly calendar (Never, Once or twice a month, Once a week, Several times a week, and Daily) because almost all U.S.-based youth use social media on a daily basis (Vogels et al., 2022). The items were worded to be comprehensible to youth from a broad age range. Examples were included in parenthesis for some items to ensure they were interpreted in the same way by all participants.

**Table 1:** Study I SASSM Initial Item Development

	Study I Item	Theme and Sub-Theme	Description from Qualitative Interviews
1	Raise awareness about a social or political issue on social media (by posting or sharing content, etc)	Restorying	Youth described raising awareness about important issues that dominant discourse (including media) were ignoring.
2	Educate others about a social or political issue on social media (by posting or sharing content, etc)	Restorying	Youth educated their followers about important sociopolitical issues. Youth made a distinction between raising awareness and educating others.
3	Debate with others about a social or political issue on social media (through a comment, post, video, etc)	Restorying	Youth engaged in debate with others on social media to counter and reframe dominant narratives.
4	Share a different perspective on U.S. history on social media than what you were taught (through a comment, post/repost, video, art, etc)	Challenging and reframing dominant narratives	A primary way youth challenged dominant narratives was by restorying historical narratives, or to include new aspects of history that were often ignored.
5	Challenge social stigmas, stereotypes, or prejudices on social media (through a comment, post/repost, video, etc)	Challenging and reframing dominant narratives	Youth challenged social stigmas, such as mental health, and prejudices, such as around body types that didn’t fit eurocentric norms, in their activism.
6	Challenge how the media covers a social or political issue on social media (through a comment, post/repost, video, etc)	Challenging and reframing dominant narratives	Youth explained that due to the media’s outsized influence on public discourse, an important way for them to change dominant narratives was by challenging the media.
7	Promote a new way of thinking or a new narrative about a social or political issue that more people need to know about (through posts/reposts, videos, art, etc)	Envisioning new futures	Although youth primarily described challenging existing narratives, a minority of youth emphasized that they participated in creating new narratives on social media (around allyship, for example).
8	Create and share art on social media about a social or political issue (paintings, drawings, music, poems, etc)	Artivism	Youth described creating art online as both important for mobilizing people around a cause or event, and for personal healing.
9	Give emotional support to others on social media (in private messages or public posts)	Emotional support	Not all youth saw emotional support as civic engagement, but those who did noted it was a powerful form of resistance online.
10	Encourage your followers to take online social or political action (by raising awareness on social media, amplifying others, attending virtual town halls, donating online, signing online petitions, using hashtags, tagging politicians, etc)	Taking collective action	Youth explicitly encouraged their followers to participate in social and political change on social media, through a variety of online methods.
11	Encourage your followers to take in-person social or political action (by attending in-person rallies, protests, blood drives, government town halls, etc)	Taking collective action	Youth used social media to mobilize their followers for in-person actions, such as protesting in-person or voting.
12	Stand in solidarity/ allyship with others on social media (by liking, sharing, or posting content)	Allyship	Youth used allyship interchangeably with solidarity, describing these actions as important aspects of building community to create change.
13	Fundraise on social media for a social or political issue	Taking collective action	Youth described fundraising, including mutual aid, as an important way to be civically engaged online.
14	Hold politicians or corporations accountable on social media for a social or political issue (by tagging them, using a hashtag, etc)	Holding people accountable	Holding people accountable was the primary way that youth described being civically engaged online for collective action, describing it as a powerful way to force people in power to listen to young people
15	Tell your personal story on social media to challenge stigmas or stereotypes (through videos, blog posts, poems, art, etc)	Restorying	Youth told their personal stories to challenge stigmas, such as certain health conditions or mental health, as well as stereotypes about their identity groups
16	Tell your personal story on social media to change how people think about a social or political issue (through videos, blog posts, poems, art, etc)	Restorying	An overaching goal that youth described in personal storytelling was to change others’ opinions and awareness about sociopolitical issues that were important to them

*Note:* This table represents the SASSM after item development was finalized, i.e., once items had been reduced and merged.

### Study II

#### Participants and Procedure

The Study II sample consisted of 809 participants recruited through Instagram advertising in July 2020 to support the validation of the SASSM. The advertisement redirected participants to an online survey hosted on Qualtrics with a consent form. The sample was majority cis-gender women (55.7%), with 35.5% cis-gender men and 8.8% non-cisgender participants, and age ranged from 14 to 25 years old (*M*_*age*_=17) with most participants (68.6%) 14–17 years old. Participants identified as Asian (19.9%), Black / African American (11.7%), Hispanic or Latinx/a/o (11.3%), White (39.9%), and Multiracial (16%), as well as American Indian / Alaska Native, Native Hawaiian or Pacific Islander, and Middle Eastern / North African (1.2%). A majority of respondents (54.5%) identified as first or second generation immigrants. Participants resided in 40 different U.S. states.

#### Study II Methods

In Study II, an exploratory factor analysis (EFA) was conducted with the 16-item scale to test its factorial structure. To determine the best-fitting factor structure eigenvalues, scree plots, and parallel analysis were utilized (El-Den et al., 2020). Factor loadings were evaluated based on a cut-off point of 0.4 (Costello & Osborne, 2005). Model fit indices were used to evaluate model fit, including the Comparative Fit Index (CFI) of ≥ 0.95, the Root Mean Square Error of Approximation (RMSEA) of ≤ 0.06, and the Standardized Root Mean Square Residual (SRMR) of ≤ 0.06 (Little, 2013).

#### Study II Results

First, data adequacy tests were conducted in SPSS version 27 using Bartlett’s test of sphericity, which confirms a significant correlation between variables, and Kaiser-Meyer-Olkin (KMO), which examines the proportion of common variance between variables. Bartlett’s test of sphericity was 0.00, under the threshold of 0.05, and KMO was 0.96, indicating that data were satisfactory for factor analysis. Next, data normality was examined. Skewness ranged from − 1.00 to 2.13, and kurtosis ranged from − 1.41 to 4.47. These tests confirmed that data were not normal, with implications for the EFA (described below). Missing data ranged from 5.30 to 6.70% for each item, with an average of 6.10% missing on each item. Using Little’s MCAR test, data were confirmed to be missing completely at random (*x*^*2*^ = 666.890, *df* = 645, *p* = .267).

Next, an EFA was conducted in MPlus version 8.1. Because data were non-normal, maximum likelihood estimation with robust standard errors (MLR) was used. Geomin Oblique was utilized because the items were expected to correlate with each other as aspects of youth online sociopolitical action. Full information maximum likelihood (FIML) estimation was used to handle missing data. The first four eigenvalues of (1) 8.058, (2) 1.247, (3) 0.948, and (4) 0.773, indicated that a two-factor solution might be best fitting. Next, the item factor loadings were investigated. In the two-factor model, only two items (items #15 and 16 in Table 2 above) loaded above 0.30 on the second factor. These two items were determined to be encompassed in items 5 and 7 (challenging stigmas, and changing how people think), and removed from the scale.

**Table 2:** Study II and Study III EFA Factor Loadings and Communalities

Study II	λ	*h* ^*2*^	Study III (Final)	λ	*h* ^*2*^
Raise awareness about a social or political issue on social media (by posting or sharing content, etc)	0.869	0.755	Raise awareness about a social or political issue on social media.	0.897	0.805
Educate others about a social or political issue on social media (by posting or sharing content, etc)	0.856	0.733	Educate your followers about a social or political issue on social media.	0.857	0.735
Debate with others about a social or political issue on social media (through a comment, post, video, etc)	0.575	0.331	Discuss or debate a social or political issue on social media.	0.661	0.437
Share a different perspective on U.S. history on social media than what you were taught (through a comment, post/repost, video, art, etc)	0.791	0.626	Share a different perspective on history than what you were taught.	0.737	0.544
Challenge social stigmas, stereotypes, or prejudices on social media (through a comment, post/repost, video, etc)	0.804	0.646	Challenge stigmas, stereotypes, or prejudices on social media.	0.832	0.692
Challenge how the media covers a social or political issue on social media (through a comment, post/repost, video, etc)	0.750	0.563	Share information or perspectives the media isn’t covering about a social or political issue.	0.840	0.706
Promote a new way of thinking or a new narrative about a social or political issue that more people need to know about (through posts/reposts, videos, art, etc)	0.822	0.675	Promote a new way of thinking or a new narrative about a social or political issue that more people need to know about.	0.817	0.667
Create and share art on social media about a social or political issue (paintings, drawings, music, poems, etc)	0.442	0.195	Create and share infographics or art (poetry, paintings, etc) about a social or political issue on social media.	0.635	0.403
Give emotional support to others on social media (in private messages or public posts)	0.504	0.254	Provide encouragement or support to others on social media.	0.677	0.458
			Tell your personal story on social media to empower others with similar identities or experiences.	0.570	.325
Encourage your followers to take online social or political action (by raising awareness on social media, amplifying others, attending virtual town halls, donating online, signing online petitions, using hashtags, tagging politicians, etc)	0.830	0.689	Encourage your followers to take social or political action (online or in-person).	0.861	0.742
Encourage your followers to take in-person social or political action (by attending in-person rallies, protests, blood drives, government town halls, etc)	0.721	0.520			
Stand in solidarity/ allyship with others on social media (by liking, sharing, or posting content)	0.603	0.363	Stand in solidarity/ allyship with others on social media.	0.775	0.601
			Amplify the voices of people who need to be heard on social or political topics.	0.874	0.764
Fundraise on social media for a social or political issue	0.442	0.295	Share links to donate on social media for a social or political cause.	0.720	0.518
Hold politicians or corporations accountable on social media for a social or political issue (by tagging them, using a hashtag, etc)	0.645	0.416	Call out injustice on social media to hold individuals or institutions accountable for their actions.	0.848	0.720

A second EFA was conducted with the 14 remaining items. The first four eigenvalues were (1) 7.407, (2) 0.953, (3) 0.861, and (4) 0.770, indicating a one-factor solution. Parallel analysis was used to determine the number of factors to retain using randomly generated correlation matrices (Hayton et al., 2004). The first two eigenvalues randomly generated were (1) 1.268 and (2) 1.206. Because the second randomly generated eigenvalue was slightly larger than the second eigenvalue, a one-factor solution was determined. Over half of the items loaded above 0.7, demonstrating that these items were satisfactory in explaining variance in the factor (see Table 2). Model fit indices for the 14-item scale suggested that a one-factor model was a good fit: the RMSEA was 0.059, the CFI was 0.951, and the SRMR was 0.035 (Little, 2013).

Before conducting Study III, the first author made three additional changes to the scale based on consultations with the second author and feedback from participants (Boateng et al., 2018). Items 10 and 11 (encouraging others to take action online and in-person) were merged to increase parsimony and reduce confusion. An open-ended question in the survey asking “Did we miss anything? Are there other ways you engage on social media for social and political issues?” resulted in two additional items on personal storytelling and amplifying marginalized voices (see Table 2 below for changes from the Study II to the Study III survey). Lastly, most examples in parentheses were removed from the items to facilitate comprehension, with the exception of artivism, as the term “art” may not be understood by all youth as inclusive of poetry, paintings, infographics, and other media.

### Study III

#### Participants and Procedure

The Study III sample consisted of 820 participants, recruited through Instagram advertising in October 2020 to validate the SASSM. Participants identified as cis-gender women (45.9%), cis-gender men (43.5%), gender nonbinary/ nonconforming (7.3%), and transgender (7.9%). Participants’ ages ranged from 14 to 25 years old (*M*_*age*_=17) with most participants (63.9%) ages 14–17. Participants identified as Asian (18.2%), Black / African American (8.1%), Hispanic or Latinx/a/o (11.7%), White (46.1%), and Multiracial (13.7%), as well as a small number of participants who identified as American Indian / Alaska Native, Native Hawaiian or Pacific Islander, and Middle Eastern / North African (2.2%). Participants who selected more than one racial and ethnic identity were coded as multiracial. Participants who did not select a category but wrote in a racial/ ethnic identity were categorized by the first author based on U.S. Census descriptions (U.S. Census Bureau, 2021). For measurement invariance tests by racial and ethnic identity, participants who identified as American Indian/Alaska Native, Native Hawaiian or Pacific Islander, and Middle Eastern/North African were removed, as low sample size precluded model estimation. A high percentage of participants (47.9%) identified as first or second generation immigrants. Respondents from 45 U.S. states were represented.

#### Study III Methods

In Study III, an EFA and CFA were conducted with the modified 15-item scale to confirm the unidimensional factor structure and to examine model consistency. An EFA and CFA are typically conducted on two independent samples (Little, 2013). Because of changes made to the SASSM between Study II and Study III, the second sample of 820 youth was randomly split into two independent samples to conduct an EFA (*n* = 250) and a CFA (*n* = 570). This approach has been used in previous psychological scale validation research (e.g., Diemer et al., 2017), and aligns with guidelines that sample sizes of 100–200 participants, or at least 10 participants per item, are sufficient for using structural equation modeling (Kline, 2015).

In Study III, an EFA was first conducted with the randomly selected sample of 250 participants to confirm the factor structure from Study II. Then, the rest of the Study III sample (*n* = 570) was utilized to conduct a CFA to evaluate model fit indices, following guidelines on goodness-of-fit cut-offs described above (Little, 2013). The same CFA sample (*n* = 570) was then used to conduct convergent validity tests with two theoretically related constructs (political efficacy and critical reflection). Finally, due to low sample sizes for certain racial and ethnic groups, the entire Study III sample (*n* = 820) was used to conduct configural, metric and scalar measurement invariance tests by age, gender, race and ethnicity, and immigrant identity. Measurement invariance determines whether items hold similar meaning for participants from different groups using a series of nested models that test for equivalent factor loadings (metric invariance) and item intercepts (scalar invariance) (Little, 2013). The configural model with no imposed constraints is compared to the metric model with factor loadings constrained to be equal across groups, and the metric model is compared to the scalar model that constrains both the factor loadings and the item intercepts to equality across groups. Theory suggests that little-to-no changes in the CFI (△CFI > 0.01), RMSEA (△RMSEA > 0.01), and SRMR (△SRMR > 0.025) demonstrate no significant decreases in model fit (Little, 2013). If any of these three fit indices were insufficient, partial invariance testing would be pursued.

#### Study III Measures

In addition to the SASSM, Study III utilized two scales to test convergent validity: critical reflection and political efficacy. A positive correlation between the SASSM scale and the two latent variables of political efficacy and critical reflection was hypothesized, supported by prior empirical research linking the three concepts (Hope et al., 2016; Diemer et al., 2017).

##### Critical Reflection

Critical reflection was measured by an 8-item sub-scale adapted from Diemer et al. 2017 (α = 0.90). The full scale has three components (Critical Reflection: Perceived Inequality, Critical Action: Sociopolitical Participation, and Critical Reflection: Egalitarianism). For this study, only the first sub-scale (Critical Reflection: Perceived Inequality) was utilized. The items assess perceived societal inequalities due to class, race, and gender, such as “Certain racial or ethnic groups have fewer chances to get a good high school education” and “Poor people have fewer chances to get good jobs.” The scale was measured on a five-point Likert scale from *strongly agree* to *strongly disagree*.

##### Political Efficacy

The Political Efficacy scale is a 4-item scale adapted from Hope and Jagers (2014), (α = 0.57) and Hope (2016), (α = 0.75). The scale measures youth perceptions of their own efficacy in improving society and creating change. The scale is coded so that higher numbers indicate greater efficacy. The items are on a five-point likert scale from *strongly agree* to *strongly disagree*, and include items such as “I believe that by participating in politics I can make a difference,” and “I have the skills and knowledge necessary to participate in politics.”

#### Study III Results

##### Exploratory Factor Analysis

A random sample of 250 youth was selected for the EFA from the Study III sample of 820 youth, leaving 570 youth for the CFA (described below). Data were not normally distributed, with most items having kurtosis less than − 1. Bartlett’s test of sphericity was significant at χ^2^(105) = 3,375.853, *p* < .001, and the Kaiser-Meyer-Olkin measure of sampling adequacy was 0.747, indicating that data were favorable for factor analysis. Missing data ranged from 4.9 to 7.2% on each item; thus FIML was utilized to use all available data. Little’s MCAR test confirmed that data were missing completely at random (*x*^2^ = 226.980, *df* = 213, *p* = .243).

Because data were not normally distributed, maximum likelihood estimation with robust standard errors (MLR) and Geomin Oblique rotation were utilized to conduct the EFA in Mplus version 8.1. Similar to Study II, the first three eigenvalues (9.484, 0.771, and 0.705 respectively) indicated that a one-factor solution might be the best fit to the data, which was further confirmed by parallel analysis using randomly generated correlation matrices (1.445, 1.343, 1.267, and 1.194). Only the first eigenvalue of 9.484 was higher than the randomly generated value of 1.445, suggesting a one-factor solution was best. The majority of the items (11 out of 15) loaded above 0.7, and all items loaded above the suggested cut-off of 0.4. All but two of the item loadings increased from the Study II to the Study III EFA, indicating that wording changes strengthened the scale.

##### Confirmatory Factor Analysis

The one-factor model in the Study III EFA was cross-validated by conducting a CFA with the random, independent sample of 570 participants to evaluate how well each item in the scale loaded onto the single factor (Kline, 2015). Maximum likelihood estimation with robust standard errors (MLR) was used because the data were not normally distributed (see Table 3). The fixed factor method was used to scale the latent construct. Model fit indices indicated the one-factor solution was a good fit to the data, well within the acceptable cut-off ranges: SRMR = 0.025, RMSEA = 0.045, and CFI = 0.975. The Chi-Square test was significant, indicating model misfit to the data, at *x*^2^ (90, *N* = 570) = 106.17, *p* = .00, yet prior research has found the Chi-Square test to be sensitive to large sample sizes and recommends prioritizing relative fit indices (Babyak & Green, 2010). Overall, the scale demonstrated a moderate to strong fit to the data, with the lowest factor loading 0.573 and the majority loading above 0.7, providing evidence that the scale aligned with this study’s conceptualization of youth sociopolitical action on social media.

**Table 3:** Final Item Study III Confirmatory Factor Loadings *(N=570)*

No.	Item	λ	SE	R^2^	Skewness	Kurtosis
1	Raise awareness about a social or political issue on social media.	.874	.014	.765	.272	-1.319
2	Educate your followers about a social or political issue on social media.	.881	.014	.776	.378	-1.285
3	Discuss or debate a social or political issue on social media.	.695	.027	.483	.492	-1.103
4	Share a different perspective on history than what you were taught.	.753	.024	.567	.653	− .864
5	Challenge stigmas, stereotypes, or prejudices on social media.	.831	.019	.691	.312	-1.352
6	Share information or perspectives the media isn’t covering about a social or political issue.	.839	.017	.705	.431	-1.171
7	Promote a new way of thinking or a new narrative about a social or political issue that more people need to know about.	.830	.016	.689	.572	− .949
8	Create and share infographics or art (poetry, paintings, etc) about a social or political issue on social media.	.643	.030	.413	.981	− .352
9	Provide encouragement or support to others on social media.	.682	.024	.465	− .251	-1.256
10	Tell your personal story on social media to empower others with similar identities or experiences.	.573	.031	.329	1.482	1.288
11	Encourage your followers to take social or political action (online or in-person).	.843	.018	.710	.571	-1.110
12	Stand in solidarity/ allyship with others on social media.	.793	.020	.628	.040	-1.477
13	Amplify the voices of people who need to be heard on social or political topics.	.854	.016	.729	.306	-1.317
14	Share links to donate on social media for a social or political cause.	.739	.021	.545	1.047	− .019
15	Call out injustice on social media to hold individuals or institutions accountable for their actions.	.857	.016	.735	.306	-1.317

*Note:* All factor loadings were significant at *p* < .001. Lambda (λ) indicates standardized factor loadings.

#### Measurement Invariance

##### Gender

Measurement invariance testing was conducted with three gender groups: cis-gender women, cis-gender men, and non-cisgender youth (including nonbinary, transgender, and youth with other gender identities combined due to low sample sizes). The configural and metric models fit well across groups (see Table 4), but changes in CFI from the metric to scalar model were higher than recommended (△CFI = 0.015). Based on modification indices, the intercepts for two items were freed for cis-gender men (Little, 2013), resulting in partial scalar invariance by gender. Intercepts were freed for Item #3: Discuss or debate a social or political issue on social media, where compared to cis-gender women (*M* = 2.54) and non-cisgender youth (*M* = 2.32) the intercepts for cis-gender men were higher (*M* = 2.99). Based on the item average of 1 standard deviation (*SD =* 1.38), these differences represented 0.33 and 0.49 *SDs*. Intercepts were also freed for Item #7: Promote a new way of thinking or a new narrative about a social or political issue that more people need to know about, where cis-gender women (*M* = 2.56) and non-cisgender youth (*M* = 2.32) had lower means compared to cis-gender men (*M* = 2.82), representing 0.19 and 0.37 *SDs* (average *SD =* 1.35). A Wald Test of parameter restraints confirmed no significant difference between the intercepts on either item for cis-gender women and non-cisgender youth.

**Table 4:** Goodness-of-Fit Indicators for Measurement Invariance by Subgroup

Subgroup	Model	*df*	x^2^	RMSEA	△RMSEA	CFI	△CFI	SRMR	△SRMR	Decision
Gender (*N* = 3 groups)	Configural	270	480.512	0.053	--	0.967	--	0.033	--	
	Metric	298	621.205	0.055	.002	0.962	.005	0.053	.019	Accept
	Scalar	326	679.436	0.058	.005	0.952	.015	0.060	.025	Reject
	Partial Scalar*	324	679.561	0.055	.002	0.958	.009	0.057	.024	Accept
Racial/ ethnic Background (*N* = 5 groups)	Configural	450	701.243	0.059	--	0.963	--	0.037	--	
	Metric	506	875.297	0.056	.004	0.962	.001	0.050	.013	Accept
	Scalar	562	920.437	0.056	.004	0.957	.005	0.054	.017	Accept
Age (*N* = 2 groups)	Configural	180	383.785	0.054	--	0.968	--	0.028	--	
	Metric	194	496.070	0.054	.000	0.964	.004	0.040	.012	Accept
	Scalar	208	532.820	0.053	.001	0.964	.004	0.041	.013	Accept
Immigrant Origin (*N* = 2 groups)	Configural	180	383.064	0.052	--	0.968	--	0.029	--	
	Metric	194	519.557	0.051	.001	0.968	--	0.033	.004	Accept
	Scalar	208	531.010	0.050	.002	0.967	.001	0.034	.005	Accept

*Note:* Chi-square calculations were completed using the scaling correction factor for MLR.

*Model freed two item intercepts for cis-gender males.

##### Racial and Ethnic Background

Next, invariance testing was conducted for youth from five racial and ethnic groups: Asian, Black or African American, Hispanic or Latino/a/x, Multiracial, and White. Fit indices for the configural model confirmed that the one factor model was a good fit. Next model comparison tests were used to confirm metric and scalar invariance (see Table 5). Scalar invariance was achieved. However, the sample size for Black and Latinx participants was under 100 for each group, and therefore results for these two groups should be interpreted with caution.

**Table 5:** Correlations Among Latent Constructs, Study III Sample *(n= 820)*

Variables	SASSM	Critical Reflection: perceived inequality
SASSM	--	
Critical Reflection: perceived inequality	.341**	--
Political Efficacy	.438**	.240 **

**p < .01

##### Age

Measurement invariance testing was conducted for two age groups as determined by literature documenting differences in youth civic engagement in high school (ages 14–17), and emerging adulthood (18–25). Fit indices confirmed that the configural model was a good fit (see Table 4). Next changes in fit indices were used to confirm metric and scalar invariance. Metric invariance was established, indicating that participants in high school and beyond interpreted the scale in similar ways. Scalar invariance was well established across both age groups.

##### Immigrant Identity

Measurement invariance testing was conducted with two groups comprised of youth of first or second generation immigrant origin (i.e., either the participant or one or both parents were born outside of the U.S.), and non-immigrant youth. Strong invariance was established across these two groups, with small changes between configural, metric, and scalar models. Thus, first and second generation immigrant and non-immigrant youth interpreted the scale similarly.

#### Convergent Validity

Next convergent validity was examined to evaluate whether the SASSM was correlated with conceptually related constructs of political efficacy and critical reflection. Using MPlus version 8.1, structural equation modeling estimated correlations among the latent variables of online sociopolitical action using the SASSM, political efficacy and critical reflection. Like the measurement invariance tests, the entire Study III sample of 820 participants was used to increase power to detect statistical significance.

As hypothesized, a significant positive correlation of 0.438 was found between the SASSM and political efficacy, higher than past studies that have reported correlations ranging from 0.21 to 0.40 (See Table 5; Hope et al., 2016). A significant positive correlation of 0.341 was found between the SASSM and critical reflection: perceived inequality, which is also slightly higher than previously documented associations with in-person actions that ranged from 0.18 to 0.29. Therefore, these findings are consistent with prior research demonstrating positive associations between youth political efficacy and critical reflection, and their online sociopolitical action. Indeed, these results show that the associations between these constructs and youth online sociopolitical action may be even greater than with their in-person sociopolitical actions.

## Discussion

In sociopolitical development theory (SPD), sociopolitical action is an important dimension of youth development whereby young people challenge and resist oppressive systems through strategic liberatory actions (Watts et al., 2011). Increasingly, young people are engaging in sociopolitical action online to challenge oppression and injustice (Anyiwo et al., 2020), yet scholarship to date has lacked a validated scale to measure youth sociopolitical action on social media. This study documented the development and validation of a scale to measure youth sociopolitical action on social media, utilizing a sequential multi-method approach including qualitative interviews to develop the scale, and quantitative surveys completed by 1,629 youth in two waves of data collection. Analyses showed evidence of validity of the scale by age, gender, racial/ethnic identity, and immigration identity, which is important for supporting its use across a range of groups.

Although prior research suggests that youth online sociopolitical action may be composed of several dimensions (Wilf & Wray-Lake, 2021), this study’s analyses concluded that a one-factor solution was the best fit for the scale. This scale may be unidimensional because digital spaces allow for greater overlap and synergy between types of sociopolitical actions. Indeed, youth may engage in multiple higher-level sociopolitical actions in a single post (Wilf et al., 2022). The scale may also be unidimensional because it measures young people’s more frequent online sociopolitical action, rather than long-term participation in coordinated organizing online. Levels of youth sociopolitical engagement may change over time, and it is important to consider the implications of sporadic versus prolonged engagement (Wray-Lake & Shubert, 2019), as these changing levels may shape other aspects of youth development, such as identity (Mathews et al., 2022) and wellbeing (Ballard & Ozer, 2016). Based on Study I interviews, the authors concluded that measuring more frequent actions was most aligned with young people’s own descriptions of their online sociopolitical action. However, a focus on more frequent behaviors may not capture the full extent of youth sociopolitical action on social media.

The scale achieved partial scalar invariance by gender by freeing the intercepts for two items for cis-gender men, related to debating and promoting new narratives. These results are compatible with prior research demonstrating that men are more likely to voice their opinions both online and offline (Brandtzaeg, 2017). Particularly online, where women (Ortiz, 2021) and transgender people (Erlick, 2018) often face targeted bullying and harassment, they may be less likely to debate with others. Overall gender differences were small, and despite these differences the SASSM provides a reasonable assessment of online sociopolitical action across genders. Further, the SASSM’s validation with both cis-gender and non-cisgender youth is important because research shows that non-cisgender youth often use social media as a space to challenge systems of oppression (Erlick, 2018).

Convergent validity tests showed that the SASSM was significantly correlated with critical reflection and political efficacy, the two other central dimensions of sociopolitical development theory. This evidence demonstrates that the SASSM is well aligned with this study’s theoretical conceptualization of sociopolitical action taken by youth online to challenge oppression. Furthermore, this study found that youth sociopolitical action on social media may be even more strongly correlated with other dimensions of sociopolitical development than their in-person sociopolitical action (see, e.g., Diemer et al., 2017; Hope, 2016), an idea to be tested in future research. Perhaps young people are better able to align sociopolitical actions with their critical reflection and political efficacy in online spaces because there are fewer barriers to online relative to in-person sociopolitical action (Marchi & Clark, 2021).

This study defined online sociopolitical action as young people’s *individual, relational, and collective efforts on digital platforms and social media to expose and rectify systems of oppression and inequity.* The authors are confident that the SASSM aligns with this definition for several reasons. First, convergent validity tests show that the SASSM is associated with the first two dimensions of SPD, critical reflection and political efficacy, indicating that the SASSM (representing the third dimension of sociopolitical action) is an integral part of youth sociopolitical development. Second, item development was based on themes inductively identified in Study I with young digital activists and modified based on feedback from young people in the Study II survey, reflecting how young people themselves refer to their own sociopolitical action online. Study I analysis was guided by an anti-oppressive lens, which is a core aspect of SPD, and most participants explicitly linked their online sociopolitical action to resisting systems of oppression. Further, the SASSM expands the SPD literature and current measures of critical consciousness (e.g., Diemer et al., 2017) by incorporating a focus on psychological forms of resistance to oppression identified in Study I, such as: challenging dominant narratives, providing emotional support to others, and telling one’s personal story to empower others with similar identities and experiences (Wilf & Wray-Lake, 2021). Although these forms of resistance are not included in current measures of sociopolitical action (e.g., Thomas et al., 2014), youth in Study I were clear that these behaviors are forms of sociopolitical action that expose and seek to rectify systems of oppression. One area that the SASSM does not measure is more formal organizing efforts youth are engaged in through social movements or organizations, which could include using social media to coordinate and plan actions. For example, organizers might use social media to communicate with other organizers to plan a tweet storm around a specific event or topic. Because a small percentage of young people are using social media for organizing at this level, the SASSM does not include items measuring these types of actions. Future research may benefit from development of a more targeted scale to measure youth organizing on social media.

This study had several strengths, including its grounding in qualitative interviews with young digital activists and its refinement based on youth feedback from the surveys. Moreover, the scale was validated with youth representing diverse gender, racial and ethnic, age, and immigrant status groups. Thus, the SASSM advances beyond other existing measures of online civic engagement by presenting evidence of validity across many groups and by using an anti-oppressive lens, which aligns with SPD. Indeed, the SASSM builds on past research on sociopolitical action by focusing on social media as the space where those actions take place (Carney, 2016), which can be used in future studies to better capture youth’s sociopolitical actions where it is happening. This scale would be useful in research that examines the extent to which processes of sociopolitical development, as well as other developmental experiences, predict youth online sociopolitical action, and how youth online sociopolitical action may relate to their in-person sociopolitical action. A primary question of interest for scholars studying youth critical consciousness and sociopolitical development is whether and to what extent youth critical reflection and political efficacy translate into critical action (e.g., Bañales et al., 2020). The SASSM could also support scholarship exploring whether and how online sociopolitical action could act as a protective factor from racism and discrimination by providing youth with a space to process and heal from negative experiences, as qualitative research suggests (Erlick, 2018). More broadly, scholars can use this scale to supplement measures of youth in-person civic engagement, resulting in a more complete and holistic view into young people’s civic lives.

Several limitations are important to note. First, Study I was conducted with young digital activists engaged in more ideologically liberal causes such as climate justice and gun violence prevention. Because of this lens, it cannot be determined whether this scale is relevant for youth who do not believe that structural oppression and discrimination exist, a limitation that is also evident in many other measures of sociopolitical action. Moreover, Study I’s qualitative sampling strategy likely did not capture all perspectives of those facing marginalization from historical and contemporary oppressions, and these experiences do not necessarily align with being politically liberal or conservative. Thus, like most existing measures of youth civic and sociopolitical engagement, the SASSM may still be somewhat ambiguous as to how the civic actor defines and conceptualizes oppression. Future work must continue to align conceptualization, measurement, and sampling in studies of resistance to oppression to add further clarity to the field.

Another limitation was that Study II and III took place at distinct sociopolitical moments that may have affected youth responses. Study II was conducted in July 2020, in the midst of COVID-19 and a surge in participation in the Black Lives Matter movement, which may have led to changes in certain kinds of youth online sociopolitical action, such as an increase in allyship (Wilf & Wray-Lake, 2021). Study III took place in October 2020, when youth contended with COVID-19 uncertainty and hybrid schooling, and may have decreased their online sociopolitical action. Researchers should continue to revisit and revise conceptualizations of online sociopolitical action to capture how it may evolve in response to future sociopolitical moments.

A final limitation was the limited racial and ethnic, gender, and non-U.S. diversity of our sample. Due to low sample sizes, non-cisgender youth were combined into a third category. This decision was intended to include all youth in analysis, but non-cisgender youth (i.e., transgender, nonbinary, and youth of other genders) should not be conflated, as they may participate in online sociopolitical action in distinct ways. Further, although measurement invariance tests showed that Asian, Black, Latinx, Multiracial, and White youth interpreted the scale similarly, measurement invariance testing by racial and ethnic identity was unable to include youth from other groups (i.e., Native American or Middle Eastern youth) due to low sample sizes. The Study III sample used for the CFA had under 100 participants for Black and Latinx youth, so results for these two racial and ethnic groups should be interpreted with caution. Third, because the entire sample for this study was U.S.-based and SPD has been studied most rigorously in the U.S. (Heberle et al., 2020), this study cannot make any determinations about whether this scale would be relevant for non-U.S.-based youth. Given that young people are increasingly taking part in online sociopolitical action around the world (Mainsah & Dralega, 2014) and youth across countries can connect and coordinate in sociopolitical action, future work that examines online sociopolitical action outside the U.S is urgently needed. Finally, although prior research has documented differences in youth social media activism by sexuality (Jenzen, 2022), this study did not ask participants about their sexuality and therefore could not conduct measurement invariance testing. Future research using the SASSM could validate the scale for youth with different sexual identities.

## Conclusion

It is increasingly important to include youth online sociopolitical action in research exploring their full civic lives, as digital spaces have become a primary context for youth sociopolitical action. The Sociopolitical Action Scale for Social Media (SASSM) developed and validated in this study was grounded in qualitative research with young digital activists and organizers, and strengthened by feedback from youth in the study. The SASSM encompasses youth individual, relational, and collective efforts to expose and rectify the root causes of systems of oppression and inequity, with an explicit focus on forms of resistance including pursuing psychological wellbeing and challenging harmful narratives. Scholars and civic educators can utilize the SASSM to address important questions regarding youth civic engagement and associations with other key developmental processes, such as critical consciousness and identity development. Ultimately, a validated scale to measure youth everyday online sociopolitical action can lead to more rigorous research and better designed programs for youth sociopolitical engagement and education, and will support the important civic work in which youth are already engaged.
